# Effect of Emotional Picture Viewing on Voluntary Eyeblinks

**DOI:** 10.1371/journal.pone.0089536

**Published:** 2014-03-05

**Authors:** Suvi Karla, Timo Ruusuvirta, Jan Wikgren

**Affiliations:** 1 Department of Psychology, University of Jyväskylä, Jyväskylä, Finland; 2 Jyväskylä Centre for Interdisciplinary Research, University of Jyväskylä, Jyväskylä, Finland; 3 Department of Psychology, University of Turku, Turku, Finland; Vanderbilt University, United States of America

## Abstract

Eyeblinks, whether reflexive or voluntary, play an important role in protecting our vision. When viewing pictures, reflexive eyeblinks are known to be modulated by the emotional state induced thereby. More specifically, the hedonic valence (unpleasantness-pleasantness) induced by the picture has been shown to have a linear relationship with the amplitude of a startle blink elicited during picture viewing. This effect has been attributed to congruence between an ongoing state and task demands: an unpleasant emotional state is assumed to bias our attention towards potentially harmful stimuli, such as startle tones. However, recent research suggests that the valence-specific modulation may not be limited to the sensory parts of the reflexive pathway related to startle responses. Here, we examined the effect of emotional picture viewing on voluntary (in response to a written command) eyeblinks in adult humans. Emotional modulation of startle blinks was also evaluated. We found that when viewing unpleasant pictures, the amplitude of reflexive eyeblinks was augmented, but the amplitude of voluntary eyeblinks was unaffected. Nevertheless, the response latencies of voluntary eyeblinks were found to be delayed during the viewing of pleasant and unpleasant relative to neutral pictures. We conclude that these results support the theory that emotional experience augments sensory processing specific to potentially harmful stimuli. Further, the emotional state seems not to exert an effect on voluntarily elicited motor activity.

## Introduction

Emotional state, particularly its hedonic valence, modulates defensive reflexes [1], [2]. The most common way to examine emotional reflex modulation is to assess the effect of emotional picture viewing on the amplitude of the eyeblink component of the acoustic startle reflex. The viewing of pictures with unpleasant content (low hedonic valence) has been found to potentiate and pictures with pleasant content (high hedonic valence) to diminish acoustic startle eyeblink reflexes. According to the motivational priming hypothesis [3], emotional picture viewing mobilizes a motivational system that, in turn, facilitates the accessibility of motor action programs that correspond to the state of this system with respect to the hedonic valence of an emotion. Reciprocally, action programs that do not correspond to the valence become less accessible and, in consequence, are less likely to be activated. Reflexes that are not inherently defensive i.e. protective, are not considered to be engaged to the motivational system and, therefore, not modulated by emotional state [4]. The modulation effect has been specifically attributed to valence. Although pictures with both high and low hedonic valence values induce heightened arousal, arousal does not affect startle amplitudes [3].

The unpleasant emotional state is assumed to bias our attention towards potentially harmful stimuli resulting in enhanced defensive responses. However, this augmentation caused by viewing unpleasant pictures has also been found for behavioral responses elicited artificially by direct stimulation of the motor cortex [5], [6]. Furthermore, such a valence-specific increase has even been observed in the accuracy of choice-reaction-time task performance through enhancement of local inhibitory networks and increased plasticity by the GABAergic system and NMDA receptors [6]. What remains an open question is whether valence-specific modulation of eyeblinks by emotional states could operate not only on the reflexive but also voluntary eyeblinks, as voluntary eyeblinks can be regarded as protective actions, even if they are not elicited by threatening stimuli.

To test this notion, we measured, in adult humans, eyeblinks that were generated either by a startle stimulus or as voluntary response to a written command.

These responses recruit the same motor mechanism (motoneurons in the facial motor nucleus or spinal cord) as that which contracts the orbicularis oculi muscle [7], [8]. However, eyeblinks are initially driven by different mechanisms, one residing in the acoustic startle pathway (reflexive eyeblinks) and the other lying in the cortex (voluntary eyeblinks). We presented the participants with unpleasant, neutral and pleasant emotional images drawn from the International Affective Picture System; IAPS [9] and measured their voluntary eyeblinks (initiated by a neutral visual stimulus, the word “BLINK”) and acoustic startle stimulus-evoked reflexive eyeblinks. We examined whether voluntary eyeblinks are affected by emotional state and, if so, whether the underlying mechanisms could be regarded as the same, or at least as analogous with the mechanisms underlying reflexive eyeblinks, in the sense that they would relate to the valence of an emotion as predicted by the motivational priming hypothesis [3].

## Material and Methods

### Participants, ethics statement and stimulus material

A total of 24 volunteers (age range 19 to 33; mean age 22, of whom 21 were females) participated in the experiment. As a compensation for their participation, the participants received a ticket to a local cinema.

This study was approved by the research ethics committee of the University of Jyväskylä and was performed in accordance with the Declaration of Helsinki.

**Figure 1:**
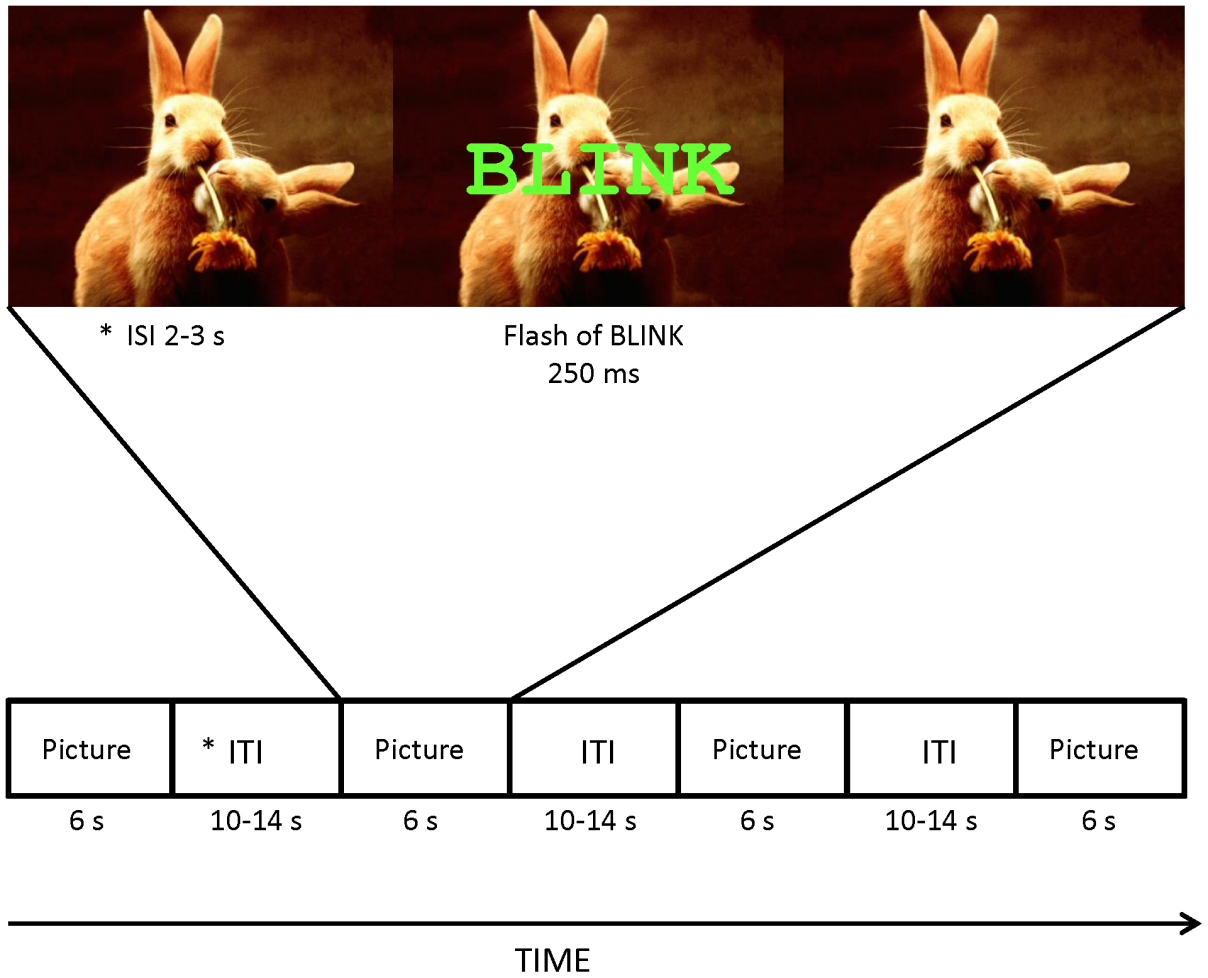
Schematic illustration of the voluntary eyeblink procedure. An example of one trial, using a pleasant picture. *ITI and ISI refer to Inter-Trial-Interval and Inter-Stimulus-Interval, respectively.

White noise startle probes (105 dB, 50 ms, center frequency 1000 Hz, 5 ms rise/fall times) were generated with SoundForge 6.0 software and presented through headphones. Voluntary eyeblinks were elicited by a visual stimulus consisting of a 250-ms flash of the word “BLINK” (lime green colour, bolded Courier New, font size 90) which appeared in the centre of the computer monitor either against a black background or superimposed on an affective picture (Figure 1). The luminosity value of each picture was analyzed with the IrfanView program to ensure the word BLINK was similarly detectable between the emotional categories. The luminosity ratings did not differ significantly between the emotional categories (pleasant, neutral, unpleasant).

The 90 emotional pictures used in the experiment were selected from the International Affective Picture System (IAPS [9]. The pictures were pleasant (e.g. food, animals etc.), neutral (e.g. household objects, neutral faces etc.) or unpleasant (e.g. mutilations, spiders etc.). The normative mean valence and arousal ratings and their standard deviations for pleasant, neutral and unpleasant pictures are reported in Table 1. These categories of pictures significantly differed from each other both in valence, i.e., pleasant vs. unpleasant [t(29) = 21.33; p<0.001], pleasant vs. neutral [t(29) = 20.36; p<0.001] and neutral and unpleasant [t(29) = 29.73; p<0.001], and in arousal, i.e., pleasant vs. unpleasant [t(29) = 9.60; p<0.001], pleasant vs. neutral [t(29) = 15.31; p<0.001] and neutral and unpleasant [t(29) = 4.07; p<0.001]. Stimulus presentation was controlled for by E-Prime 2.0 software.

**Table 1:** The normative mean valence and arousal ratings and their standard deviations.

IAPS pictures	Pleasant	Neutral	Unpleasant
The normative mean (SD) valence ratings	7.53(0.43)	5.04(0.60)	2.49(0.71)
The normative mean (SD) arousal ratings	4.90(1.02)	2.87(0.42)	6.01(0.89)
The mean (SD) luminosity ratings	111.20(41.39)	101.52(36.65)	97.66(39.11)

Orbicularis oculi EMG activity was recorded from beneath the left eye by using two disposable Ag/AgCl electrodes, following the guidelines presented in [10]. The active electrode was attached vertically below the pupil and was referenced to an electrode about 1 cm lateral to it. The EMG data were recorded with a QuickAmp amplifier using BrainProducts Recorder software running on a PC. All off-line signal processing was conducted using Matlab with the Signal Processing Toolbox.

## Procedures and Experimental Design

Upon arrival at the laboratory, participants were instructed to read and, if they decided to participate, sign an informed consent form which provided general information on the experiment. During the experiment, the participants were seated in a comfortable chair in a dimly lit room with a 17-inch computer monitor located approximately 1 m in front of them. They were instructed to view the pictures shown on the computer monitor and to ignore all irrelevant stimuli in the experimental room. During the experiment, occasional tones or the flash-word BLINK would be presented. Randomly selected examples of the BLINK stimuli both on the affective picture and on the black background were presented to the participants before the experiment. The experiment consisted of two procedures: a startle eyeblink procedure and a voluntary eyeblink procedure. The order of the two procedures was counterbalanced across the participants.

### The startle eyeblink procedure

The startle eyeblink procedure consisted of two phases: a habituation phase and an acquisition phase. In the habituation phase, 5 startle tone-alone trials were presented in order to familiarize the participants with the loud tone. In the acquisition phase, 30 trials with the startle tone and randomly selected affective picture content (pleasant, neutral or unpleasant, 10 each), 10 trials with the startle tone alone and 9 trials with affective pictures alone (3 neutral, 3 pleasant and 3 unpleasant) were presented in pseudo-random order. The participants were informed that they would hear occasional tones, but their task was to ignore them.

### The voluntary eyeblink procedure

In the voluntary eyeblink procedure, 30 trials with randomly selected affective picture content (pleasant, neutral or unpleasant, 10 each) and the visual stimulus, 10 trials with the visual stimulus alone (black background) and 9 trials with affective pictures alone (3 neutral, 3 pleasant and 3 unpleasant) were presented in pseudo-random order. The participants were informed that their task was simply to blink their eyes once every time they saw the word “BLINK”.

During the startle and voluntary eyeblink procedures, the pictures were visible for 6 seconds and the startle tone or the word BLINK was presented either 2 or 3 s from image onset. The inter-trial interval, during which the display was black, was randomly either 10, 12 or 14 s.

## Data Analysis

The startle and voluntary blink EMG signal was amplified, digitized (sampling rate 2000 Hz), rectified and digitally off-line filtered with a low-pass (<20 Hz) filter. First, bad trials were visually excluded with the observer blind to the trial type. The exclusion criterion was excessive EMG activity during the 500-ms baseline period. Peak amplitude and the peak and onset latencies over the period 20 to 150 ms after startle tone onset were determined automatically with a Matlab script. The values were then averaged for each type of picture content (pleasant, neutral and unpleasant).

The same measures were determined for the voluntary eyeblink responses. Visually identified bad trials were first excluded by an observer blind to the trial type. The exclusion criteria were i) excessive EMG activity during the 500-ms baseline period or a blink the onset latency of which occurred during the first 100 ms after the BLINK stimulus, and ii) no response up to 1 000 ms after the BLINK stimulus. Onset latencies were visually determined trial-by-trial on a computer display and hand-scored. Peak amplitudes and latencies were determined automatically with a Matlab script for the period of 100 to 1 000 ms after the BLINK stimulus. One participant was discarded owing to very late responses (mostly in excess of 1 000 ms after the BLINK stimulus).

Analysis of variance for repeated measures and additional post-hoc paired samples *t*-tests with Bonferroni-correction were used to analyze the effects of conditioning and image content. Greenhouse-Geisser-corrected degrees of freedom were used if the sphericity assumption was violated.

## Results

### Peak amplitudes, onset latencies and peak latencies

**Figure 2:**
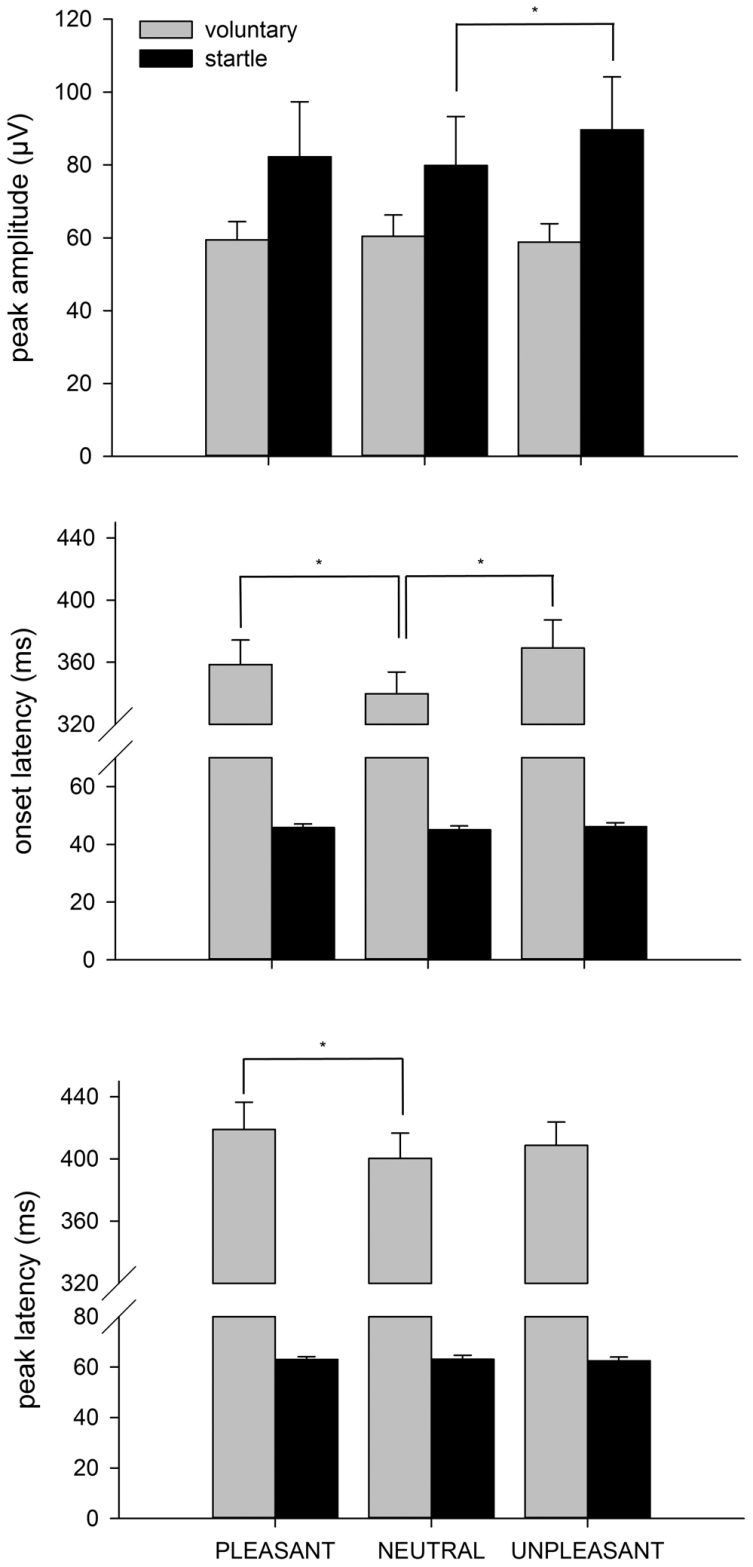
Startle and voluntary eyeblink properties (+ SEM) during affective picture viewing, *p<0.05. The amplitudes of the startle eyeblinks were significantly augmented during the unpleasant picture viewing. No such effect on the amplitude of the voluntary eyeblinks was observed. The onset latencies for both pleasant and unpleasant images were longer than those for neutral images and the voluntary eyeblinks during the viewing of pleasant images had significantly longest peak latency.

The peak amplitudes in both eyeblink types (startle vs. voluntary) as a function of image content (pleasant, neutral, unpleasant) were analysed with a 2×3 repeated measures ANOVA, which showed a significant interaction between factors [F(2,44) = 3.36; p<0.05], indicating that the modification effect of image content on blink amplitude was different between the response types. The main effect of response type was also significant [F(1,22) = 4.70; p<0.05], indicating larger peak amplitudes in startle responses than in voluntary blinks. ANOVA performed for each response type separately revealed a significant main effect of image on startle peak amplitude [F(2,46) = 3.23; p<0.05], indicating that emotional picture viewing modified the startle eyeblinks (Figure 2). Pairwise comparisons between pleasant vs. unpleasant [t(23) = 1.61; p = 0.122] and pleasant vs. neutral [t(23) = 0.59; p = 0.559] did not reach significance, but the difference between neutral and unpleasant was significant [t(23) = 3.08; p<0.01], indicating that the largest startle eyeblinks were elicited during unpleasant picture viewing. For voluntary eyeblinks, no main effect of image on peak amplitude was observed [F(2,44) = 0.44; p = 0.647].

Repeated measures ANOVA for a main effect of image content and response type on onset latency showed significant interaction [F(2,44) = 5.89; p<0.01], indicating that image content had a different modulatory effect on the timing of startle blinks compared to voluntary blinks. Both response and image type had a significant main effect [F(1,22) = 425.92; p<0.001 and F(2,44) = 7.06; p<0.01, respectively]. ANOVAs performed for each response type separately showed no significant main effect of image type on onset latency for startle blinks but a main effect was found for voluntary blinks [F(2,44) = 6.62; p<0.01]. Subsequent paired t-tests showed that the onset latencies for both pleasant and unpleasant images were longer than those for neutral images [t(22) = 2.38, p<0.05 and t(22) = 3.18, p<0.01], respectively (Figure 2).

Image x response type ANOVA revealed an almost significant interaction [F(2,44) = 2.41, p = 0.10] and main effect of image type [F(2,44) = 3.09, p = 0.056]. Response type had a clearly significant effect [F(1,22) = 508.40, p<0.001]. ANOVAs performed for each response type separately revealed no significant effect of image type on peak latency for startle blinks [F(2,46) = 0.23, p = 0.793] and a nearly significant effect for voluntary blinks [F(2,44) = 2.74, p = 0.076]. Subsequent paired t-tests indicated that voluntary blinks during the viewing of pleasant images had a significantly longer peak latency than those during the viewing of neutral images [t(22) = 2.38, p<0.05] (Figure 2).

## Discussion

The present study investigated whether emotional picture viewing similarly affects the amplitude of voluntary and reflexive eyeblinks. In the case of reflexive eyeblinks, the highest amplitude was found with the viewing of unpleasant pictures. In contrast, no such valence effect was found in the amplitude of voluntary eyeblinks. The only effect of emotional picture viewing on voluntary eyeblinks was in onset latency which was longer with pleasant and unpleasant pictures relative to neutral pictures.

The augmentation of reflexive eyeblinks by unpleasant pictures was highly expected (e.g. [1], [2]) and is in line with the motivational priming hypothesis [3]. However, no linear trend was observed in startle eyeblink modification, because smallest responses were measured during neutral picture viewing. The reason for this is unclear, but one possibility may be, that even if the IAPS picture series is widely used in several cultures, Finnish participants' experience of the pleasant pictures may differ from e.g. those of North American. In fact, the same pattern was observed in our previous study on the effect of emotional picture viewing on the amplitude of conditioned eyeblink responses [11].

The modification of startle eyeblinks is a phenomenon which is believed to index defensive-protective tendencies. The simplest and fastest pathway for reflexive eyeblinks that is modulated by emotional valence is the acoustic startle pathway. This pathway consists of cochlear root neurons which project to neurons in the nucleus reticularis pontis caudalis, and motoneurons in the facial motor nucleus (pinna reflex) and spinal cord [12]–[14]. The nucleus reticularis pontis caudalis receives projections from the central nucleus of the amygdala [14], [15]. These projections have an important role in mediating fear/defense responses, and are likely to mediate the valence effects of reflexive eyeblinks. The central nucleus of the amygdala also has reciprocal connections to various cortical sites [15], implying that valence-specific reflex modification, as allowed by the amygdala, may also apply to cortically-induced behavioral responses, such as voluntary eyeblinks As both of these mechanisms are accessible by the central nucleus of the amygdala, we reasoned that if valence-specific emotional modulation is not restricted to the reflex generating mechanism, such modulation should be similarly observed in both modes of eyeblink responses. The results, however, did not show this to be the case.

The negative valence of the pictures could be regarded as facilitating the mechanism for reflexive eyeblinks of a defensive nature. In contrast, voluntary eyeblinks did not show similar augmentation. Although these responses are initially driven by different mechanisms, they are both accessible by the central nucleus of the amygdala [15], and thus can be expected to show emotional modulation. However, as voluntary eyeblinks are also mediated by cortical connections, they are generated later than reflexes and are most probably affected by “cognitive appraisal”. Therefore, even if the motor output is the same, the present results imply that the valence-specific emotional modulation observed might be more restricted to the reflex generating mechanism and not affect the voluntary eyeblinks. It is possible that emotional state modulates only those actions that are initiated by biologically meaningful threat signals that reach the amygdala. Information about voluntary actions might reach the amygdala differently or to lesser extent, which might explain the lack of emotional modulation of voluntary eyeblinks.

However, voluntary eyeblinks were affected by the emotional picture viewing, as they were significantly delayed during both pleasant and unpleasant images in comparison to neutral images. This effect could be due to either arousal or, more simply, to the fact that the emotion-evoking pictures tend to be more complex. Thus, the relative delay induced by emotional pictures in voluntary eyeblinks may relate to selective attention [2]. Namely, attentional mechanisms might have prioritized pictures inducing high emotional arousal over the written “BLINK” command as an emotionally neutral stimulus, thereby delaying the execution of the voluntary blink. This possibility is further supported by previous findings where behavioral responses to a neutral stimulus were delayed by background stimuli of high emotional arousal [16], [17].

It might also be suggested that valence played no observable role in modifying the amplitude of voluntary eyeblinks because the visual stimulus inducing these blinks (the word “blink”) was not of a clearly threatening nature (for the congruency assumption of the motivational priming hypothesis, see [3]). This is, however, not supported by previous findings of responses induced by TMS pulses to the motor cortex augmented by unpleasant relative to pleasant or neutral pictures [5], [6], as TMS pulses, directly delivered to the motor cortex, cannot per se be considered as perceptually threatening stimuli. This possibility could, nevertheless, be tested by also making the visual stimulus itself indirectly threatening (for example by using a word that signals a threat, such as “air puff”, that can be removed by a blink). If such a setting fails to produce valence-specific effects, then it is possible that emotional valence can only modify a behavioral response that is both defensive in nature and an innate reaction to a threat cue.

The sensitivity of reflexive but not voluntary eyeblinks to emotional valence was unlikely to be accounted for by the different stimulus modalities (visual vs. auditory) used to induce them, as startle eyeblinks can be similarly amplitude-modulated by emotions irrespective of whether the eyeblinks are induced by visual, acoustic or tactile stimuli [18], [19]. In fact, it would be possible to examine the stimulus modality effect by using an auditory instead of visual command to elicit the voluntary eyeblink and so test whether the voluntary eyeblink remains be non-sensitive to emotional valence.

To conclude, we found augmentation of reflexive eyeblinks to unpleasant pictures and delayed voluntary eyeblinks to both unpleasant and pleasant pictures relative to neutral pictures. This pattern of findings suggests that emotional experience augments sensory processing that is specific to potentially harmful stimuli and that in terms of their sensitivity to emotional states, reflexive and voluntary eyeblinks recruit different mechanisms as described by the motivational priming hypothesis.

## Supporting Information

Appendix S1 (DOCX)
