# Supplementary material for: Effect of Emotional Picture Viewing on Voluntary Eyeblinks
Source: PLoS One. 2014 Mar 5;9(3):e89536. doi: 10.1371/journal.pone.0089536 (PMC3943727; doi:10.1371/journal.pone.0089536)
Supplement: Appendix S1 — (DOCX) [file pone.0089536.s001.docx]

The IAPS pictures were:

Pleasant: 1440, 1460, 1600, 1603, 1610, 1620, 1750, 1920, 2040, 2050, 2080, 2091, 2208, 2250, 2331, 4650, 4660, 4680, 5200, 7270, 7280, 7330, 7350, 8030, 8080, 8120, 7200, 7230, 8200, 8510

Neutral: 5500, 5510, 7000, 7010, 7020, 7030, 7050, 7060, 7080, 7090, 7100, 7110, 7150, 7170, 7500, 5900, 7004, 7184, 7211, 7217, 7233, 7130, 7180, 2514, 5740, 5750,7002, 7009, 7031, 7705

Unpleasant: 2120, 3000, 3010, 3030, 3120, 3130, 3140, 3150, 6200, 6230, 9040, 9050, 1120, 1274, 1280, 1300, 2095, 2683, 3530, 6370, 9490, 2710, 2800, 6415, 6555, 9001, 2053, 2141, 7380, 9041
